# Consistency and inconsistency in network meta-analysis: model estimation using multivariate meta-regression‡

**DOI:** 10.1002/jrsm.1045

**Published:** 2012-07-20

**Authors:** Ian R White, Jessica K Barrett, Dan Jackson, Julian PT Higgins

**Affiliations:** a MRC Biostatistics UnitCambridge, UK; b Centre for Reviews and Dissemination, University of YorkUK

## Abstract

Network meta-analysis (multiple treatments meta-analysis, mixed treatment comparisons) attempts to make the best use of a set of studies comparing more than two treatments. However, it is important to assess whether a body of evidence is consistent or inconsistent. Previous work on models for network meta-analysis that allow for heterogeneity between studies has either been restricted to two-arm trials or followed a Bayesian framework. We propose two new frequentist ways to estimate consistency and inconsistency models by expressing them as multivariate random-effects meta-regressions, which can be implemented in some standard software packages. We illustrate the approach using the mvmeta package in Stata. Copyright © 2012 John Wiley & Sons, Ltd.

## Introduction

Network meta-analysis (NMA) in its standard form makes an assumption of ‘consistency’ (Lu and Ades, 2006), also called ‘coherence’ (Lumley, 2002), which means that estimates of treatment effects from direct and indirect evidence are in agreement, subject to the usual variation under the random-effects model for meta-analysis. Consider the case with three treatments A, B and C. If trials comparing B with A, C with A, and C with B estimate parameters *δ*^*AB*^, *δ*^*AC*^ and *δ*^*BC*^, respectively, then consistency means that *δ*^*AB*^ + *δ*^*BC*^ = *δ*^*AC*^: the effect of B relative to A, plus the effect of C relative to B, equals the effect of C relative to A.

The consistency assumption is often questionable. For example, the trials that do not include treatment A may include a population for whom A is inappropriate and hence, their results may differ systematically from trials that do include A (Song *et al*., 2009). Alternatively, trials that include A may be older and therefore use different implementations of other treatments. Wrongly assuming consistency can lead to misleading conclusions, so procedures to investigate the possibility of inconsistency are important.

Some authors have proposed ways to assess consistency directly from fitting consistency models (Dias *et al*., 2010, Lu *et al*., 2011). This paper instead assesses consistency by fitting both consistency and inconsistency models. The companion paper (Higgins *et al*., 2012) reviews the meaning of inconsistency and methods for assessing it (Salanti *et al*., 2008; Lu and Ades, 2006; Lumley, 2002) and argues that inconsistency is best modelled by a design-by-treatment interaction. We follow that paper in using *design* to refer only to the set of treatments compared in a trial.

Models for consistency and especially inconsistency are complex. Lumley (2002) proposed a frequentist approach that was valid when all trials had only two arms. Lu and Ades (2006) proposed a Bayesian approach that accommodated multi-arm trials and could be implemented in WinBUGS software (Lunn *et al*., 2000). This paper proposes two new computational strategies for estimating consistency and inconsistency models when the data include multi-arm trials. The new strategies differ from the Bayesian approach in two ways. First, they are frequentist methods, so they aim to speed up computation, avoid sensitivity to the choice of priors and avoid Monte Carlo error. Second, they are two-stage estimation procedures, unlike the one-stage Bayesian procedure. Both strategies formulate the consistency and inconsistency models as multivariate random-effects meta-regressions which can now be easily fitted in standard software (White, 2009, 2011).

The paper is set out as follows. We first introduce data from an NMA of thrombolytic drugs and introduce multivariate meta-analysis and meta-regression in general terms. We then formulate a model for NMA allowing for heterogeneity and inconsistency and describe our new proposals for two simple estimation methods and the alternative Bayesian analysis. We compare the three methods using the thrombolytic drugs data and end with a discussion.

## Example: thrombolytic drugs

As an example, we use a dataset consisting of 28 trials comparing eight thrombolytic treatments after acute myocardial infarction: streptokinase (A), accelerated alteplase (B), alteplase (C), streptokinase plus alteplase (D), tenecteplase (E), reteplase (F), urokinase (G) and anti-streptilase (H) (Lu and Ades, 2006). The data are shown in Table 1. Two of the studies are three-arm trials and the rest are two-arm trials. The usefulness of NMA is emphasised by examples such as this, where many pairs of treatments are not compared head to head in any trial. For example, the only way to assess the effect of reteplase (F) relative to urokinase (G) is by utilising the indirect evidence from trials in designs 5 and 6 which compare F and G with A and from designs 9 and 10 which compare F and G with B, although design 12, which compares G with C, also contributes to the indirect evidence. We aim to re-analyse these data to explore and test for consistency, and if consistency is not rejected, to rank the treatments.

**Table 1:** The thrombolytic drugs data: entries are numbers of deaths in 30 or 35 days/number of patients. Bold entries show designs where inconsistency parameters are introduced (see text)

Design *d*	Study	Streptokinase (A)	Accelerated alteplase (B)	Alteplase (C)	Streptokinase + alteplase (D)	Tenecteplase (E)	Reteplase (F)	Urokinase (G)	Anti-streptilase (H)
1	1	1462/20 173	652/10 344		723/10 328				
2	2	1455/13 780		1,418/13 746					1448/13 773
3	3	9/130		**6/123**					
	4	5/63		**2/59**					
	5	3/65		**3/64**					
	6	887/10 396		**929/10 372**					
	7	7/85		**4/86**					
	8	12/147		**7/143**					
	9	10/135		**5/135**					
4	10	4/107			**6/109**				
5	11	285/2992					270/2994		
6	12	10/203						7/198	
7	13	3/58							**2/52**
	14	3/86							**6/89**
	15	3/58							**2/58**
	16	13/182							**11/188**
8	17		522/8488			523/8461			
9	18		356/4921				**757/10 138**		
	19		13/155				**7/169**		
10	20		2/26					**7/54**	
	21		12/268					**16/350**	
11	22		5/210						**17/211**
	23		3/138						**13/147**
12	24			8/132				**4/66**	
	25			10/164				**6/166**	
	26			6/124				**5/121**	
13	27			13/164					**10/161**
	28			7/93					**5/90**

## Multivariate random-effects meta-regression

Suppose each study yields estimates of *p* different quantities of interest: in this paper, these will be different between-treatment contrasts, but in other applications of multivariate random-effects meta-regression, they may represent different study outcomes (Jackson *et al*., 2011). We write these *p* estimates for study *i* as the (1 × *p*) vector **y**_*i*_. We assume we also know **S**_*i*_, the ‘within-study’ (*p* × *p*) variance–covariance matrix of **y**_*i*_. The meta-regression model is


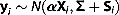
(1)

where **X**_*i*_ is a (*q* × *p*) design matrix containing the covariates (White, 2011). In this model, the unknown parameters are ***α***, a (1 × *q*) vector of regression coefficients and **Σ**, a (*p* × *p*) between-studies variance–covariance matrix, assumed the same for all studies.

Usually, the different quantities in **y**_*i*_ have separate regressions, with the *j*th quantity regressed on *q*_*j*_ covariates. Then, the mean of the *j*th quantity is modelled as ***α***_*j*_**x**_*ij*_, where ***α***_*j*_ is a (1 × *q*_*j*_) vector and **x**_*ij*_ is a (*q*_*j*_ × 1) vector. In this case, 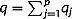 and (1) holds with


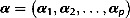
(2)

and


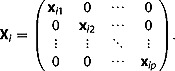
(3)

The multivariate meta-analysis model is the special case of (1) with **X**_*i*_ equal to the (*p* × *p*) identity matrix, so that


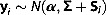
(4)

and ***α*** is a (1 × *p*) vector of the overall means of the estimates (White, 2009; Jackson *et al*., 2011). The standard meta-regression model (Thompson and Sharp, 1999) is the special case of (1) with *p* = 1, so that **y**_*i*_, **S**_*i*_ and **Σ** are scalars.

Multivariate random-effects meta-analysis is implemented in Stata as mvmeta (White, 2009), which has recently been extended to implement multivariate random-effects meta-regression (White, 2011). Restricted maximum likelihood (REML) estimation is usually used for **Σ** to avoid the negative bias associated with maximum likelihood estimation of variance components. Likelihood-based estimation can straightforwardly accommodate studies in which some elements of **y**_*i*_ are missing.

## NMA model formulation

In this section, we state a model assuming that one of the treatments, which we call treatment A, is included in every trial. This is rarely true in practise and is not true for the thrombolytic drugs data. For the alternative case where the trials have no common treatment, we later describe different modifications and show how they can be expressed by the multivariate meta-regression model (1).

We consider a network including a total of *T* treatments A, B, C, and so on. Let *d* = 1, …, *D* index the designs (the sets of treatments compared in a trial). Let there be *n*_*d*_ trials of the *d*th design, each comparing *T*_*d*_ treatments. Thus, in Table 1, *d* = 1 corresponds to the design comparing A, B and D; this is a three-arm design, so *T*_1_ = 3, and only one such trial is present, so *n*_1_ = 1.

Salanti *et al*. (2008) compared ‘arm-based’ models describing arm-specific parameters, such as the log odds, and ‘contrast-based’ models describing contrasts of arm-specific parameters, such as log odds ratios. We initially use a contrast-based approach.

Let 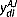 be the observed contrast of treatment J (J = B, C, …) with treatment A in the *i*th trial (*i* = 1 to *n*_*d*_) in the *d*th design (*d* = 1 to *D*). 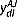 may represent any measure, such as a mean difference, a standardised mean difference, a log risk ratio or a log odds ratio. Our model for the observed data is


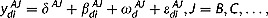
(5)

where *δ*^*AJ*^ represents a contrast (a summary effect) between J and A, 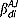 represents heterogeneity in the J–A contrast between studies within designs, 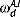 represents inconsistency in the J–A contrast (heterogeneity between designs), and 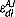 is a within-study error term. Equivalently, in vector notation,


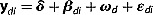
(6)

where 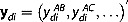, ***δ*** = (*δ*^*AB*^, *δ*^*AC*^, …)′, 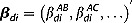, 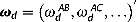 and 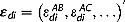. For the within-study error terms, we assume ***ε***_*di*_ ∼ *N*(0, **S**_*di*_) where **S**_*di*_ is assumed to be known. The treatment contrasts ***δ*** are regarded as fixed parameters. We consider the specification of the heterogeneity terms ***β***_*di*_ and the inconsistency terms ***ω***_*d*_ below.

For treatments J not used in design *d*, 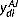 is missing. This is not a problem; we simply use the model implied by (6) for the observed subvector of **y**_*di*_.

### Modelling heterogeneity

Heterogeneity, represented by the 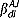 terms, refers to variation between true treatment effects in trials of the same design. We regard it as a random effect


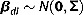
(7)

as in the conventional random-effects model for meta-analysis. We refer to model (7) without constraint on **Σ** as the unstructured model. In this model, the between-studies variance is 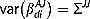 for the J–A contrast and 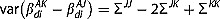 for the K–J contrast (J,K ≠ A); between-studies variances of different contrasts are distinct. *Σ* is identified under consistency if every pair of treatments has been compared in at least two studies, but estimation is typically very imprecise with small numbers of studies.

We now describe two possible structured heterogeneity models for **Σ**, which are more readily identified (Salanti *et al*., 2008; Lu and Ades, 2009).

In a fully structured heterogeneity model, we assume that all treatment contrasts have the same between-studies variance *τ*^2^. By considering the between-studies variance of the J–A contrasts, it follows that *Σ*^*JJ*^ = *τ*^2^ for all J. By considering the between-studies variance of the K–J contrasts, it also follows that *Σ*^*JK*^ = *τ*^2^/2 for J ≠ K. Thus,


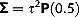
(8)

where **P**(*ρ*) is a matrix with all diagonal entries equal to 1 and all off-diagonal entries equal to *ρ*.

Partially structured heterogeneity models are also possible. For example, the choice **Σ** = *τ*^2^**P**(*ρ*) for unknown *ρ* might be useful, because it implies that all treatment contrasts involving treatment A have variance *τ*^2^ and all treatment contrasts not involving treatment A have a different variance 2(1 − *ρ*)*τ*^2^.

### Modelling inconsistency

Inconsistency is represented by the 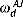 terms. These could be treated as random effects with mean zero (Lumley, 2002; Lu and Ades, 2006) or as fixed effects (Lu and Ades, 2006). In the companion paper, we argue that they are best treated as fixed effects (Higgins *et al*., 2012). Here, we have an additional practical reason for treating them as fixed effects; if we treated them as random effects, then model (6) would have three variance components (within-study, between-study and inconsistency) so could not be expressed in the meta-regression framework (1).

We model inconsistency using the design-by-treatment interaction model described in the companion paper. This specifies no structure for the 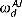 terms. Thus, each treatment contrast is allowed to differ freely across designs; for example, the B–A contrast in trials with design AB can differ from that in trials with design ABD. Inconsistency models with fewer parameters are possible, such as the model of Lu and Ades (2006), which depends on a particular ordering of the treatments (Higgins *et al*., 2012).

Only a limited number of 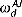 parameters is usually needed in the model; for example, 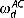 is not needed in design AB. In the design-by-treatment interaction model, the number of identifiable 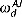 parameters (the number of degrees of freedom (DOF) for inconsistency, *df*_inc_) is the difference between the number of identified fixed parameters in the design-by-treatment interaction model, ∑ _*d*_(*T*_*d*_ − 1), and the number of identified fixed parameters in the consistency model, *T-1*, so *df*_inc_ = ∑ _*d*_(*T*_*d*_ − 1) − (*T* − 1).

There are various equivalent ways to parameterise the model. A simple approach, illustrated below, considers the designs in order. It adds a parameter for inconsistency in any design which includes a pair of treatments whose contrast can be estimated either directly from a previous design or indirectly under the consistency model from two or more previous designs. For multi-arm designs, the number of inconsistency parameters added to a design depends on how many of its treatment contrasts can be estimated from previous designs.

Below we show two ways to estimate models (6) and (7) with fixed inconsistency parameters using a two-stage approach.

### Testing consistency

Once an inconsistency model has been fitted, using the methods to be described below, it is possible to test the hypothesis of consistency. This is best performed by globally testing all the inconsistency parameters using the global Wald test statistic 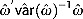 which under consistency follows a *χ*^2^ distribution on *df*_inc_ DOF. Because we use REML estimation, likelihood ratio tests are not available for comparing models with different covariates. Different parameterisations for the models – different choices of the reference treatment or different ways to construct the inconsistency parameters – may be used, but the global Wald test should give the same results for all parameterisations.

It is also possible to examine and test individual *ω* parameters. However, it is important to remember that a different parameterisation – for example, a different choice of reference treatment – would lead to a different interpretation. This point is illustrated below.

Like any global test, the global Wald test may lack power. Thus, when the hypothesis of consistency is not rejected, inconsistency may nevertheless be present. It is important to use one's understanding of the nature and design of the studies in the NMA to decide how plausible is consistency. If large inconsistency is plausible, then the NMA should be broken down – by restricting the treatments or the trials – into a smaller problem where consistency is more plausible. If the hypothesis of consistency is not rejected and is plausible on subject-matter grounds, then it may be reasonable to base inferences on the consistency model.

If the significance test rejects the hypothesis of consistency, then treatment effects estimated from the consistency model are especially suspect. Instead, it is important to try to understand the source(s) of inconsistency, just as the sources of heterogeneity should be explored in standard meta-analysis (Thompson, 1994). Examination of the 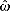 parameters may help here. Possible explanations should be sought in terms of differences in study-level covariates such as the date of the study or the nature of the population (Salanti *et al*., 2009). If successful, these covariates could be included in the NMA model or used to stratify the analysis. Another possible explanation is that particular studies are outliers. If outliers are also of inferior methodological quality, then they might be excluded from the analysis, but if they are of equal or superior quality, then there is no alternative to a careful understanding of the nature and reliability of the data.

## Estimation by the standard approach

We now consider model estimation where no treatment is common to all trials. The standard contrast-based model (Salanti *et al*., 2008) designates *trial-specific* reference treatments. For example, sensible choices of reference treatment in the data of Table 1 would be A for designs 1–7, B for designs 8–11 and C for designs 12–13. Contrasts of non-reference treatments with the reference treatment and their standard errors are then estimated.

First, consider the case when only two-arm trials are present. Define 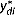 as the single estimated treatment contrast in trial *i* of design *d*. If design *d* compares treatments J and K, then model (6) implies


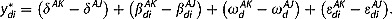
(9)

We now describe how this model can be expressed as a standard univariate meta-regression (model (1) with *p* = 1), following Salanti *et al*. (2008).

The first term in (9) involves fixed parameters and enters the ***α*****X**_*i*_ term of model (1) by constructing covariates 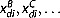 for each treatment except A. For the JK design, the covariate for K is 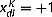, the covariate for J is 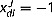, and all other covariates 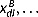 are 0.

The second term in (9) is a random variable representing heterogeneity. Under the unstructured heterogeneity model (7), its variance is *Σ*^*JJ*^ − 2*Σ*^*JK*^ + *Σ*^*KK*^, which varies between designs. Under the fully structured heterogeneity model (8), its variance is *τ*^2^ in all designs. To use the meta-regression framework (1), which imposes a common between-studies variance for all studies, we must therefore assume the fully structured heterogeneity model (8).

The third term, representing inconsistency, involves fixed parameters and requires covariates constructed carefully as described above. Lumley (2002) proposed a similar model for NMAs comprising only two-arm trials with the inconsistency parameters as random effects.

The fourth term is a random variable, representing within-study error in the K–J contrast, and its variance can be estimated directly from the study-level data.

Now, consider the case with three-arm trials; each yield two estimated treatment contrasts with within-study covariances assumed known. Standard univariate meta-regression software is not now appropriate and to date, only Bayesian estimation has been described (Lu and Ades, 2006). We now express this case in the multivariate meta-regression framework (1).

Define 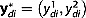 as the set of estimated contrasts in three-arm trial *i* of design *d*. For design *d* comparing treatments J, K and L, the expectation of 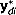 under consistency is *δ***X**_*di*_, where *δ* = (*δ*^*AJ*^, *δ*^*AK*^, *δ*^*AL*^) and 
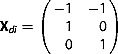
. In general, each outcome is regressed on *T-1* covariates constructed to equal −1 for the reference treatment, + 1 for the treatment compared with the reference and 0 for the other treatments. The coefficients *δ* are the same for all outcomes, so the structure differs from that in equations (2) and (3).

With this reformulation, the model can be fitted by a two-stage procedure using the Stata programme mvmeta (White, 2009, 2011) and the code in Appendix A.1. Imposing common coefficients across outcomes requires a previously unpublished option commonparm which was implemented for this paper. Extension to trials with four or more arms is straightforward.

## Estimation by data augmentation

We now propose an alternative two-stage approach for data that include multi-arm trials; here, the same treatment is taken as the reference treatment in all designs. We accommodate designs which do not include the reference treatment by a data augmentation technique, which introduces an artificial reference treatment arm containing a very small amount of information. Data augmentation is a computational device to enable easy model estimation in a two-stage framework. The advantages of this approach over the standard approach are that model (6) can be estimated directly and intuitively; modelling and estimation are simplified; and more flexible modelling of heterogeneity, as in equation (7), is possible.

We use an arm-based approach to motivate the data augmentation approach in the contrast-based model. We take arm A as the reference category in all trials, irrespective of their design. For trials without arm A, we augment the data by introducing a very small amount of information in arm A. If the trial outcome is binary, the augmenting information is *h* individuals with success fraction *m*, for a small value of *h*. In the analyses below, we use *h* = 0.001 and set *m* equal to the overall event fraction pooling all treatments and all trials (0.08 in the thrombolytics data). Sensitivity to choices of reference treatment, *h* and *m* is explored below. If the trial outcome is quantitative, the augmenting information could be a mean equal to the overall mean and a standard error *M* ≫ 1 times the largest arm-specific standard error.

Table 2 shows why the augmentation method works, using studies 2, 6 and 27 from the thrombolytics data which compare three treatments A, C and H. Study 2 includes all three treatments and hence yields estimates of both contrasts, which are assembled into the vector **y**_*di*_ with the variance–covariance matrix **S**_*di*_ shown. Both variances in **S**_*di*_ are small, because there is information about both contrasts. The **X**_*di*_ matrix shows that **y**_*di*_ estimates the two basic parameters. Study 6 includes A and C but not H, so it only yields an estimate for the first component of **y**_*di*_; the second component is missing, and correspondingly, **S**_*di*_ has just one non-missing element, and **X**_*di*_ has just one non-missing column. Study 27 includes C and H but not the reference treatment A. For the standard approach, **y**_*di*_ has just one non-missing component corresponding to the H–C contrast. The **X**_*di*_ matrix shows that it estimates the difference of the two basic parameters. For the data augmentation approach, an arm A is introduced. The estimated **S**_*di*_ contains very large variances, representing the lack of information in this trial about the C–A and H–A contrasts. However, the equally large covariance in ***S**_di_* means that the information about the H–C contrast is correctly conveyed; setting *c* = (−1, 1)^*T*^, the H–C contrast is **c**^*T*^**y**_*di*_ = − 0.262 with variance **c**^*T*^**S**_*di*_**c** = 0.19, the same as with the standard approach. The ***X***_*di*_ matrix shows that **y**_*di*_ estimates the two basic parameters as in study 2.

**Table 2:** Subset of the thrombolytic drugs data (deaths/patients), showing coding for the standard and data augmentation approaches. Parameter vector is *δ* = (*δ*^*AC*^, *δ*^*AH*^)

Study	A	C	H	**y**_*di*_	**S**_*di*_	**X**_*di*_
2	1455/13 780	1418/13 746	1448/13 773	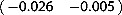	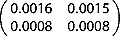	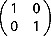
6	887/10 396	929/10 372	.	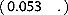	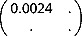	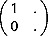
27, Standard	.	13/164	10/161	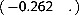	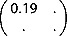	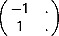
27, Data augmentation	0.00008/0.001	13/164	10/161	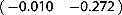	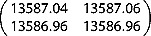	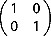

In the case of binary outcomes, zero counts in observed treatment arms require additional treatment; the standard procedure is to add 1/2 to all cells in observed treatment arms for any trial with a zero count (Sweeting *et al*., 2004). A better but more computationally demanding, the way to handle binary data is to use the full binomial likelihood (Hamza *et al*., 2008).

### Fitting the models to the augmented dataset

The consistency model (equation (5) with all *ω* terms equal to zero) can be written as


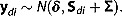
(10)

This is a multivariate random-effects meta-analysis as in equation (4). With the *ω* parameters as fixed effects, the inconsistency model can be written as a multivariate random-effects meta-regression


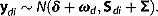
(11)

The random effects in such a model represent heterogeneity within designs. For trials missing one or more of arms B, C and so on, **y**_*di*_ is incomplete, but likelihood-based procedures naturally accommodate this.

### Ranking in the consistency model

If the consistency model is supported by the data, then it is useful to report the probability that each treatment is the best treatment (Caldwell *et al*., 2005). This is most naturally performed in the Bayesian approach but can also be approximated in the frequentist approach using a parametric bootstrap procedure implemented in mvmeta (White, 2011). We describe the procedure in the case where positive elements of ***δ*** identify treatments that are worse than the reference treatment. Parameter vectors 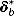, for *b* = 1, …, *B*, are drawn from the approximate posterior 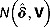, where 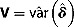. For each *b*, the best treatment is identified as treatment A if all elements of 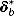 are positive and otherwise as the treatment corresponding to the lowest element of 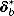. The probability that each treatment is best is estimated by the fraction of the *B* draws for which that treatment was best. Other summaries, such as rankograms or the probability that each treatment is the worst (Salanti *et al*., 2011) can be estimated similarly but are usually of less clinical interest.

As described, the method ranks the overall treatment effects ***δ***; the predicted treatment effects in a new trial, ***δ*** + ***β***_*di*_, can be compared by instead drawing 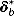 from 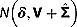 (Higgins *et al*., 2009; Riley *et al*., 2011).

## Estimation by the Bayesian approach

We also consider a Bayesian version of our model, which we implement for the thrombolytics data in WinBUGS. This model uses binomial within-study distributions in a one-stage analysis and does not require data augmentation or any approximation. We use an arm-based approach, where for each trial, we model a baseline treatment outcome *μ*_*di*_ and other treatment outcomes as comparisons to the baseline treatment. For convenience, we directly model the treatment comparison in each design, 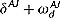, and reparameterise to obtain results for the inconsistency parameters 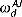. A Bayesian version of the Wald test statistic for inconsistency can be computed. Alternatively, consistency and inconsistency models can be compared using the deviance information criterion (Spiegelhalter *et al*., 2002).

We use prior distributions for modelling the baseline treatment outcomes, *μ*_*di*_ ∼ *N*(0, 100), and treatment comparisons, 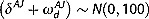. If **Σ** is unconstrained, a Wishart prior could be used for **Σ**^− 1^. For the constrained model (8), an appropriate prior distribution is *τ* ∼ *U*(0, 2). Results may be sensitive to the prior distribution for the heterogeneity parameters (Lambert *et al*., 2005).

For each WinBUGS run, we used a burn-in of 30 000 updates. Convergence was checked using the Gelman–Rubin statistic as modified by Brooks and Gelman (1998), calculated for three chains with different initial parameter values. Every 20th update was sampled to reduce auto-correlation. Results were calculated from 150 000 sampled updates, ensuring that the estimated Monte Carlo error for all parameters was less than 0.005.

## Results for thrombolytics data

Stata code for the frequentist analyses of the thrombolytics data is given in Appendix A, and WinBUGS code for the Bayesian analysis is given in Appendix B.

### Choice of heterogeneity model

Table 1 shows that only eight contrasts are found in more than one trial, and only seven contrasts are found in more than one trial of the same design. The arguments above show that we cannot estimate an unstructured heterogeneity model. Instead, we fit the fully structured heterogeneity model (8), which assumes the same heterogeneity variance between each pair of treatments. The partially structured heterogeneity model Σ = *τ*^2^P(*ρ*) gives restricted log likelihoods that vary by no more than 0.02 as *ρ* varies from 0 to 1 (results not shown).

### Consistency model

The consistency model (Table 3) shows that treatments B, E and G are most likely to be the best, with similar results for REML and Bayesian estimation. Point estimates were similar between the two estimation methods, except that the estimated heterogeneity was very small for REML but larger for Bayesian estimation. Standard errors were larger for the Bayesian analysis because of the larger estimated heterogeneity and because Bayesian analysis better allows for uncertainty in the heterogeneity parameter.

**Table 3:** Thrombolytic drugs data: results from consistency and inconsistency models. ‘REML’ is the data augmentation approach using ***h* = 0.001, *m* = 0.08** and with 10 000 parametric bootstrap samples to compute P(best). ‘Bayes’ is the Bayesian approach and estimates are posterior means

		Consistency model				Inconsistency model	
			
		Estimate (standard error)		P(best)		Estimate (standard error)	
				
Treatment	Parameter	REML	Bayes	REML	Bayes	REML	Bayes
A	-			0.00	0.00		
B	*δ*^*AB*^	−0.16 (0.05)	−0.23 (0.14)	0.19	0.17	−0.16 (0.22)	−0.16 (0.31)
C	*δ*^*AC*^	0.00 (0.03)	−0.02 (0.10)	0.00	0.01	−0.03 (0.22)	−0.03 (0.31)
	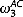					−0.16 (0.32)	−0.18 (0.38)
D	*δ*^*AD*^	−0.04 (0.05)	−0.06 (0.14)	0.00	0.02	−0.04 (0.22)	−0.04 (0.31)
	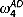					0.45 (0.73)	0.48 (0.82)
E	*δ*^*AE*^	−0.16 (0.08)	−0.22 (0.22)	0.23	0.28	−0.15 (0.32)	−0.15 (0.45)
F	*δ*^*AF*^	−0.11 (0.06)	−0.18 (0.16)	0.07	0.11	−0.06 (0.23)	−0.06 (0.32)
	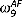					−0.18 (0.40)	−0.21 (0.52)
G	*δ*^*AG*^	−0.20 (0.22)	−0.23 (0.24)	0.51	0.41	−0.35 (0.55)	−0.37 (0.60)
	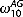					0.33 (0.71)	0.38 (0.80)
	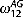					0.05 (0.69)	0.05 (0.77)
H	*δ*^*AH*^	0.01 (0.04)	0.04 (0.11)	0.00	0.04	−0.00 (0.22)	−0.00 (0.30)
	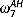					−0.06 (0.41)	−0.06 (0.47)
	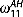					1.20 (0.53)	1.25 (0.64)
	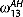					−0.31 (0.45)	−0.32 (0.52)
Heterogeneity	*τ*	0.02 (0.08)	0.12 (0.10)			0.22 (0.14)	0.26 (0.15)
Wald test of consistency ( 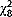 )						8.61	7.91
Deviance information criterion			95.92				97.96

### Inconsistency model

For these data, the design-by-treatment interaction model has 15 fixed parameters (two for each of the two three-arm designs and one for each of the 11 two-arm designs), and the consistency model has seven fixed parameters. There are thus 15 − 7 = 8 DOF for inconsistency.

We parameterise the design-by-treatment inconsistency model by working down the designs in Table 1. Bold font in Table 1 indicates designs to which we attach inconsistency parameters. Designs 1 and 2 have no potential for inconsistency. Design 3 introduces potential for inconsistency because the C–A contrast can differ between designs 2 (ACH) and 3 (AC); we call this ‘design inconsistency’ in the companion paper (Higgins *et al*., 2012). We therefore attach an inconsistency parameter 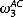 to arm C in design 3. We similarly attach inconsistency parameters to D in design 4 and H in design 7. Design 9 introduces potential for inconsistency in a different way, because it directly estimates the F–B contrast which is indirectly estimated under consistency by combining designs 1 (ABD) and 5 (BF); we call this ‘loop inconsistency’ in the companion paper (Higgins *et al*., 2012). We therefore attach an inconsistency parameter 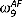 to arm F in design 9. We similarly attach inconsistency parameters to G and H in designs 10–13 because the contrasts in these designs (BG, BH, CG and CH) are all estimable under consistency from earlier designs.

Table 3 reports results for REML estimation via data augmentation and for Bayesian estimation. The REML Wald test for inconsistency was 8.60 on 8 DOF, showing no overall evidence for inconsistency. The Wald-like statistic for the Bayesian analysis was similar at 7.80. The deviance information criterion was lower for, and hence shows greater support for, the consistency model.

In the design-by-treatment inconsistency model (Table 3), seven of the eight inconsistency parameters are smaller than their standard errors, but 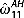 is slightly more than twice its standard error. Given the multiple testing and the overall lack of evidence for inconsistency, this is likely to be a chance finding. However, to illustrate interpretation as in Table 5 of the companion paper (Higgins *et al*., 2012), we now take 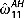 at face value; it represents a discrepancy between the direct estimate of the H–B contrast from design 11 and the indirect estimate from the other designs, especially designs 1 (AB) and 2 and 7 (AH), suggesting inconsistency around loop ABH. Indeed, design 11 seems to show large benefit of B over H, whereas designs 1, 2 and 7 show little difference between A, B and H.

Different parameterisations can lead to different conclusions about individual parameters but not overall. For example, if we parameterise the design-by-treatment inconsistency model by working up, instead of down, the designs in Table 1, then the overall test for inconsistency changes by less than 0.001 (code in Appendix A.2, results in Table 4). However, now, two parameters (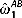 and 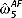) are larger than twice their standard errors. Large 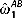 suggests inconsistency around loop ABH, as with the first parameterisation, whereas 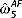 suggests a second form of inconsistency which was not seen with the first parameterisation.

**Table 4:** Thrombolytic drugs data: results from two different parameterisations of the inconsistency model, using the data augmentation approach with *h* = 0.001, *m* = 0.08

		Parameterisation 1		Parameterisation 2	
			
Treatment	Parameter	Estimate	(Standard error)	Estimate	(Standard error)
A	-				
B	*δ*^*AB*^	−0.16	(0.22)	−1.42	(0.56)
	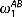			1.26	(0.60)
C	*δ*^*AC*^	−0.03	(0.22)	0.22	(0.52)
	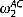			−0.25	(0.56)
	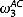	−0.16	(0.32)	−0.41	(0.57)
D	*δ*^*AD*^	−0.04	(0.22)	0.41	(0.69)
	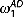			−0.45	(0.73)
	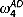	0.45	(0.73)		
E	*δ*^*AE*^	−0.15	(0.32)	−1.41	(0.60)
F	*δ*^*AF*^	−0.06	(0.23)	−1.51	(0.60)
	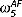			1.45	(0.64)
	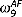	−0.18	(0.40)		
G	*δ*^*AG*^	−0.35	(0.55)	−0.05	(0.63)
	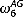			−0.30	(0.84)
	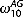	0.33	(0.71)	−1.23	(0.79)
	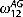	0.05	(0.69)		
H	*δ*^*AH*^	−0.00	(0.22)	−0.06	(0.35)
	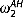			0.06	(0.41)
	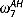	−0.06	(0.41)		
	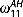	1.20	(0.53)		
	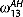	−0.31	(0.45)		
Heterogeneity	*τ*	0.2156	(0.1445)	0.2156	(0.1445)
Wald test of consistency ( 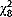 )		8.6053		8.6050	

Surprisingly, the estimated heterogeneity is much larger in the inconsistency model than in the consistency model. Because the contrasts between designs contribute to the heterogeneity in the consistency model and not in the inconsistency model, this may arise because one or more contrasts between designs are smaller than expected by chance. Univariate analysis shows that the C–A contrast, which probably contributes most information about heterogeneity, is very similar between designs 2 (AC) and 3(ACH).

It is possible to fit other inconsistency models using our framework, as described in the companion paper (Higgins *et al*., 2012).

### Choice of reference categories, *h* and *m*

Table 5 explores sensitivity of the data augmentation approach to different choices of the parameters *h* and *m* and different choices of reference category. The results of the Wald test of consistency are very similar in all cases except that mild differences are seen with *h* = 0.1 and *m* = 0.5. Estimates of treatment contrasts AB and AG showed excellent agreement in all cases. Similar comparisons for the other treatment contrasts showed even closer agreement (results not shown). In the data augmentation approach, we calculated matrices **S**_*di*_ using double precision computation (that is, with accuracy to about 16 significant figures); by contrast, using single precision (that is, with accuracy to about eight significant figures), agreement between approaches was poorer and computation failed for *h* < 0.001.

**Table 5:** REML estimation of thrombolytic drugs data: Wald *χ*^2^ statistics testing consistency, selected estimated treatment effects and their standard errors, comparing standard approach with data augmentation approach for various choices of *h* and *m* and the reference treatment

		Data augmentation approach							
		
	Standard approach	Reference treatment	m	0.08			0.5		
					
			h	0.001	0.01	0.1	0.001	0.01	0.1
Wald test of consistency 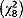	8.61	A		8.61	8.61	8.60	8.60	8.59	8.42
		B		8.61	8.61	8.62	8.61	8.62	8.64
		C		8.61	8.61	8.60	8.60	8.59	8.35
Treatment effect *δ*^*AB*^	−0.161	A		−0.161	−0.161	−0.161	−0.161	−0.161	−0.162
		B		−0.161	−0.161	−0.161	−0.161	−0.161	−0.158
		C		−0.161	−0.161	−0.161	−0.161	−0.161	−0.162
Treatment effect *δ*^*AG*^	−0.197	A		−0.197	−0.197	−0.197	−0.197	−0.198	−0.205
		B		−0.197	−0.197	−0.198	−0.197	−0.198	−0.202
		C		−0.197	−0.197	−0.197	−0.197	−0.198	−0.202
Standard error se(*δ*^*AB*^)	0.046	A		0.046	0.046	0.046	0.046	0.046	0.046
		B		0.046	0.046	0.046	0.046	0.046	0.046
		C		0.046	0.046	0.046	0.046	0.046	0.047
Standard error se(*δ*^*AG*^)	0.222	A		0.222	0.222	0.222	0.222	0.222	0.221
		B		0.222	0.222	0.222	0.222	0.222	0.221
		C		0.222	0.222	0.222	0.222	0.222	0.221

These results confirm that the choice of reference category is unimportant, support the argument for taking *m* to be the observed mean of the data and show that *h* = 0.001 was a good choice in these data.

## Discussion

We have demonstrated two frequentist estimation procedures for consistency and inconsistency models for NMA. Our procedures are approximate in two ways. First, they are two-stage procedures which summarise the data from each study as a point estimate and variance, so they implicitly approximate the within-study log-likelihood by a quadratic function of the parameters. This is typically a good approximation except with very sparse data, that is, where many study arms have no events (or no non-events). Second, the data augmentation approach approximates by adding a small amount of information in the reference category. The comparisons in Table 5 shows that this is an excellent approximation.

The alternative Bayesian procedure avoids both of these approximations, but it has its own difficulties. First, it is computationally intensive; fitting the inconsistency model took about 2 h for the Bayesian procedure and 8–14 s for each frequentist procedure on a Windows Server running on a multi-user Sun computer with 2.59-GHz processors. Second, results can be very sensitive to the prior distribution chosen for the heterogeneity parameters.

The two frequentist procedures fit the same basic model but have different strengths and weaknesses. The disadvantage of the data augmentation approach is the extra approximation involved. Its advantages are (i) the between-studies variance matrix *Σ* need not follow the fully structured model (8), unlike in the standard approach, and (ii) the modelling framework is more intuitive, with treatment effects appearing as overall means rather than as coefficients of design variables. To allow treatment effects to vary with a study-level covariate W, the data augmentation approach enters W as a covariate, whereas the standard approach requires the interaction between W and the design variables to be entered in the model.

If the data augmentation approach is used for binary outcomes, *m* should be the observed mean and *h* should take a small value such as 0.001. Although the choice of reference category should not affect the model fit, it may be convenient in terms of interpretation to use an untreated control or placebo group as reference. Alternatively, computational speed might be improved by using a treatment found in many designs as reference category. Finally, when substantial data augmentation has been performed, it may be wise to check that results are similar between different choices of the reference treatments, *h* and *m*.

Although we have illustrated the methods using Stata, other software can be used. In R, the data augmentation approach can be implemented using the mvmeta package (Gasparrini, 2011). In SAS, both frequentist approaches can be implemented using proc mixed (van Houwelingen *et al*., 2002), and a referee has pointed out that the models can also be fitted in a one-stage procedure using conditional logistic regression (Stijnen *et al*., 2010).

Future research should explore the interpretation of individual parameters in the design-by-treatment interaction model; the power to test for inconsistency in this model; possible ways to assess inconsistency by posterior predictive checks; possible reduced inconsistency models with fewer DOF and greater power under specific forms of inconsistency; and partially structured models for heterogeneity.

Network meta-analysis is increasingly popular. This paper has contributed a frequentist analysis approach based on multivariate meta-regression, which should make analysis easier and faster in the presence of multi-arm trials, gives a global test for consistency, and is able to rank the treatments in a way that was formerly only possible with a Bayesian analysis.

## Appendix A Code for frequentist analysis in Stata

The code below uses data with one record per study, with identifiers design and study. Numbers of events/individuals are r1/n1 for treatment 1 (A), and so on, up to r8/n8 for treatment 8 (H). mvmeta may be installed by typing net install mvmeta, from(http://www.mrc-bsu.cam.ac.uk/IW_Stata/meta) in Stata.

### Appendix B Code for Bayesian analysis in WinBUGS

This code fits the inconsistency model to the thrombolytics data and should be easily adaptable to other examples. The main data are arranged with one record per arm: d and study indicate which design and study that arm belongs to, t indicates its treatment, and b indicates the first treatment in that design. r and n are the numbers of successes and individuals in the arm. The supplementary data offset and offset.design list the rows in which the first arm of each trial and of each design is found. S = 28 is the number of studies, T = 8 is the number of treatments, A = 58 is the total number of treatment arms in all trials, and D = 13 is the number of designs.

To simplify the WinBUGS code, we use a reparameterised version of the model (6). We re-express *δ* and *ω*_*d*_ as a *D*x*T* matrix eff.des: for each design, eff.des[d,t]  is the summary treatment effect of treatment t relative to the baseline treatment of that design, allowing for inconsistency; for treatments not in the design, corresponding elements of eff.des are undefined. For each study i, eff.study[i,t,b]  is the true effect comparing treatment t with b for that study, allowing for heterogeneity. Because the data are expressed in this way, the inconsistency parameters w and one of the treatment effects dAE (for which no direct comparison is available) must be computed indirectly.

The consistency model can be fitted by replacing eff.des[d,k] with eff.des[k] and eff.study[i,t[k],b[k]] with eff.study[i,k].
